# Health Care Provider Communication: An Empirical Model of Therapeutic Effectiveness

**DOI:** 10.1002/cncr.27949

**Published:** 2013-01-22

**Authors:** Harvey M Chochinov, Susan E McClement, Thomas F Hack, Nancy A McKeen, Amanda M Rach, Pierre Gagnon, Shane Sinclair, Jill Taylor-Brown

**Affiliations:** 1 Department of Psychiatry, University of ManitobaWinnipeg, Manitoba, Canada; 2 Manitoba Palliative Care Research Unit, CancerCare ManitobaWinnipeg, Manitoba, Canada; 3 Patient and Family Support Services, CancerCare ManitobaWinnipeg, Manitoba, Canada; 4 Faculty of Nursing, University of ManitobaWinnipeg, Manitoba, Canada; 5 Psychology Department, University of MemphisMemphis, Tennessee; 6 Faculty of Pharmacy, Laval UniversityQuebec City, Quebec, Canada; 7 Spiritual Care Services, Tom Baker Cancer CenterCalgary, Alberta, Canada

**Keywords:** communication, psychosocial, distress, empirical, model

## Abstract

**BACKGROUND:**

Patients who are facing life-threatening and life-limiting cancer almost invariably experience psychological distress. Responding effectively requires therapeutic sensitivity and skill. In this study, we examined therapeutic effectiveness within the setting of cancer-related distress with the objective of understanding its constituent parts.

**METHODS:**

Seventy-eight experienced psychosocial oncology clinicians from 24 health care centers across Canada were invited to participate in 3 focus groups each. In total, 29 focus groups were held over 2 years, during which clinicians articulated the therapeutic factors deemed most helpful in mitigating patient psychosocial distress. The content of each focus group was summarized into major themes and was reviewed with participants to confirm their accuracy. Upon completion of the focus groups, workshops were held in various centers, eliciting participant feedback on an empirical model of therapeutic effectiveness based on the qualitative analysis of focus group data.

**RESULTS:**

Three primary, interrelated therapeutic domains emerged from the data, forming a model of optimal therapeutic effectiveness: 1) *personal growth and self-care* (domain A), 2) *therapeutic approaches* (domain B), and 3) *creation of a safe space* (domain C). Areas of domain overlap were identified and labeled accordingly: domain AB, *therapeutic humility*; domain BC, *therapeutic pacing*; and domain AC, *therapeutic presence*.

**CONCLUSIONS:**

This empirical model provides detailed insights regarding the elements and pedagogy of effective communication and psychosocial care for patients who are experiencing cancer-related distress. [See editorial on pages 000–000, this issue.] Cancer 2013. © 2013 American Cancer Society.

## INTRODUCTION

Distress is a significant problem for individuals who are living with cancer. Estimates of substantial emotional distress in these patients range from 20% to 50%.^1^-^6^ Distress is often diverse, tainting physical, psychosocial, existential, and spiritual aspects of patient experience. It can include feelings of hopelessness, dependency, loss of control, uncertainty; worrying about the future, concern about being a burden to others, and loss of dignity.^7^-^12^ Although poor communication with health care providers is associated with suffering,^13^-^16^ quality psychosocial care can alleviate distress and improve patients' quality of life.^9^, ^17^, ^18^

Despite its pivotal role in patient care, there is limited information about the constituents of therapeutic effectiveness in the setting of cancer-related distress. This limitation makes it challenging to describe or effectively teach clinicians how to provide optimal psychosocial care. Although training guidelines for psychosocial clinicians^19^-^21^ identify basic counseling competencies,^22^ there are few empirical models integrating the multitude of expert clinical skills needed to assuage the distress of patients living with cancer.^23^ Various studies have examined core competencies of effective therapists^24^-^26^; however, these have tended to rely on very small samples of expert informants, have addressed basic competencies for trainees, and have not focused on medical populations.^27^, ^28^

In this second phase of a 2-part study on cancer-related distress,^29^ we examined the elements of therapeutic effectiveness. Highly experienced Canadian psychosocial oncology clinicians were invited to take part in a series of focus groups, eliciting detailed descriptions of how they communicate with and approach distressed patients with cancer. Ethical approval was received from the Health Research Ethics Board at the University of Manitoba and from 15 additional review boards covering all academic or clinical sites in which participants held affiliations.

## MATERIALS AND METHODS

### Participant Selection

The principal investigator contacted 14 directors of departments of psychosocial oncology located within major Canadian cancer centers. These directors were provided a description of the study; in turn, they identified 24 additional directors/supervisors, who were asked to inform their professional psychosocial staff about the study. Clinicians were eligible to participate if they were engaged in psychosocial work with cancer patients in any capacity and had the support of their supervisor. This approach resulted in 90 clinicians from 24 health centers in 21 cities across Canada enrolling in the first phase of the study.^29^ Seventy-eight (ie, 87%) of those clinicians took part in the second phase of the study, the results of which are reported herein. Upon completion of the series of focus groups, in-person workshops were held across Canada, eliciting participant feedback on a model of therapeutic effectiveness based on focus group data.

### Data Collection Procedures

All participants provided informed consent and basic demographic information. Between March 2009 and February 2011, clinicians attended 3 focus groups approximately 6 months apart. To facilitate rich and meaningful discussions, centers were video-linked with 1 to 3 other study sites based on time zone and telemedicine server-issue considerations. The principal investigator, a senior clinician, and the project manager—both based at the Manitoba Palliative Care Research Unit—facilitated each focus group. All meetings—29 in all—were 2 to 2.5 hours in length and were audio taped and transcribed.

During the focus groups, clinicians were invited to discuss and share their reflections regarding all aspects of “how they address patient distress in their therapeutic encounters.” They were asked to share clinical experiences and to describe in detail what takes place during their time with patients. These discussions often addressed the qualities they brought that facilitated patient disclosure and how they understood and managed their responses to these encounters.

### Data Management and Analysis

The project manager and principal investigator identified key themes arising from each focus group. Summaries listing the themes, illustrated with focus-group exemplars, were then distributed to all focus group participants. The principal investigator reviewed these at the outset of their next group meeting, hence providing an effective means for *member checking*, a strategy used to help ensure the rigor of qualitative research.^30^ This process confirmed the trustworthiness of the data and facilitated a rapid re-entry into discussions regarding clinicians' responses to patient distress.

At the end of the study, 4 coders (H.M.C., S.E.M., T.F.H., and N.A.M.) reread all of the group summaries and analyzed the data using content analysis and constant comparative techniques.^31^-^34^ Through discussion and consensus building, the 4 coders were able to reduce 155 initial themes to 47 broader, summative themes. (Two additional themes, v*alue professional development* [Table 1, item 6] and *assure confidentiality* [Table 1, item 25] were added on the basis of study workshop feedback.) Definitions were then written for each of the themes and major categories. In the next round of analysis, the researchers sought agreement on how these themes fit into a larger framework. By examining commonalities and differences between these themes and overarching schemas, an empirical model of therapeutic effectiveness emerged.

**TABLE 1:** The Model of Therapeutic Effectiveness: Domains and Themes^a^

Primary Domains	Overlapping or Hybrid Domains
A: *Personal growth and self-care*	AB: *Therapeutic humility*
1. Maintain a balanced life	26. Do not avoid emotion
2. Work at self-awareness	27. Tolerate clinical ambiguity
3. Acknowledge/work through our own fears	28. Be able to explore difficult topics
4. Acknowledge your own feelings of vulnerability or helplessness	29. Accept and honor client as expert
5. Debrief with colleagues	30. Be a catalyst for therapeutic change
6. Value professional development	31. Trust in the process
B: *Therapeutic approaches*	32. “Sit with” client emotional distress
7. Clarify and name sources of distress	33. Avoid urge to have to fix
8. Problem-solve	34. Model healthy processing of emotion
9. Educate, inform client	BC: *Therapeutic pacing*
10. Debunk myths	35. Listen attentively
11. Reinforce client strengths and positive ways of coping	36. Hold or ground client
12. Provide techniques (eg, mindfulness, Therapeutic Touch)	37. Keep client in the here and now
13. Advocate for client with the care team	38. Maintain slow pace—do not rush therapy
14. Foster positive relationships between client and family	39. Encourage client to talk about fear and distress
15. Elicit client needs	40. Normalize and validate client experience and distress
16. Probe for feelings underlying events and circumstances	41. Use skillful tentativeness, ie, be “purposefully hesitant” to be nonthreatening
17. Help client identify what they can and cannot control	AC: *Therapeutic presence*
18. Help client understand by mirroring and reflection	42. Being compassionate and empathetic
19. Use silence to encourage client expression	43. Being respectful and nonjudgmental
20. Explore image and metaphor	44. Being genuine and authentic
21. Offer comfort through touch	45. Being trustworthy
22. Acknowledge spiritual distress	46. Being fully present
C: *Creation of a safe space*	47. Valuing intrinsic worth of client
23. Provide privacy	48. Being mindful of boundaries
24. Provide calming environment	49. Being emotionally resilient
25. Assure confidentiality	ABC: *Optimal therapeutic potential*
	50. By skillfully combining elements contained within each of the domains, clinicians are able to achieve optimal therapeutic effectiveness

a For a schematic of the model, see Figure 1.

At the end of the study, face-to-face workshops were held in 8 cities across the country (with clinicians being asked to travel to their nearest center). Participants attending these workshops were provided an opportunity to assess the emerging model of therapeutic effectiveness and discuss the content, meaning, and wording of each model item. Participants also were administered a questionnaire, which asked them to evaluate the overall model and critique the placement of each individual theme.

## RESULTS

Participants included 50 social workers (64%); 8 physicians (11%), 6 psychologists (8%), 5 nurses (6%), 5 spiritual care providers (6%), and 4 other counselors (5%). They had an average of 17.5 years of professional experience and 8.5 years in psychosocial oncology (see Table 2). On average, they saw 5 new patients per week within their health care setting, hospital inpatient/outpatient unit, or in hospice. Eleven clinicians withdrew from the study because of changing jobs (n = 5), being too busy to participate (n = 3), taking extended leave (n = 1), retirement (n = 1), or withdrawing for unknown reasons (n = 1). Sixty-four of 78 clinicians (82%) attended 1 of the final workshops.

**TABLE 2:** Demographic and Professional Description of Participants

Characteristic	No. of Participants (%)^a^	No. of Participants (%)^b^
Sex		
Women	61 (78)	49 (78)
Men	17 (22)	14 (22)
Highest education		
Bachelor's degree/college	16 (20)	9 (14)
Master's degree	48 (62)	42 (67)
MD/PhD	14 (18)	12 (19)
Marital status		
Married	59 (76)^c^	48 (77)
Divorced/separated	8 (10)	6 (9)
Never married	10 (13)	9 (14)
Profession		
Social work	50 (64)	41 (65)
Medicine	8 (11)	7 (11)
Psychology	6 (8)	4 (6)
Spiritual care	5 (6)	5 (8)
Nursing	5 (6)	3 (5)
Other health care	4 (5)^d^	3 (5)^e^
Years in profession: Mean±SD	17.5±10.3	17.1±9.3
Years in oncology: Mean±SD	8.5±8.0^c^	8.1±7.5

Abbreviations: SD, standard deviation.

a These were 78 participants who attended at least 1 of 3 focus groups and contributed to the data from which the model was developed. Percentages indicate the proportion of participants who were included in at least 1 focus group in each category.

b These were 63 participants who attended the final meeting in which the model was assessed. Percentages indicate the proportion of participants at the final meeting in each category.

c Of 78 participants, 1 did not report marital status, and 1 did not indicate the number of years spent in psychosocial oncology.

d Other health care professions included occupational therapists (n = 2) and clinical counselors (n = 2).

e Other health care professions included occupational therapists (n = 1) and clinical counselors (n = 2).

The model of therapeutic effectiveness is comprised of 3 primary domains—*personal growth and self-care*, *therapeutic approaches*, and *creation of a safe space* (see Fig. 1, domains A, B, and C, respectively) and 3 overlapping or hybrid domains (*therapeutic humility*, *therapeutic pacing*, and *therapeutic presence* (see Fig., domains AB, BC, and AC, respectively).

**Figure 1:**
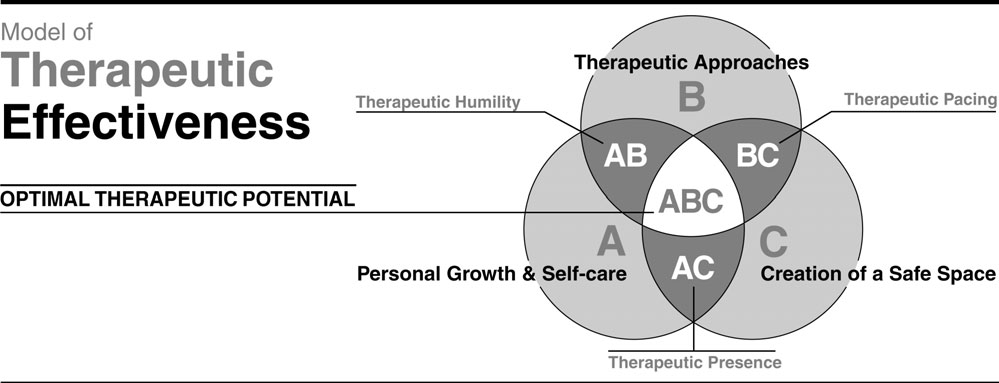
The model of therapeutic effectiveness is comprised of 3 primary domains (domain A, *personal growth and self-care*; domain B, *therapeutic approaches*; and domain C, *creation of a safe space*) and 3 overlapping or hybrid domains (domain AB, *therapeutic humility*; domain BC, *therapeutic pacing*; and domain AC, *therapeutic presence*). Domain ABC indicates optimal therapeutic effectiveness (see Table 1).

### Primary Domains

#### Domain A: Personal growth and self-care

This domain is comprised of 6 themes, each referring to various elements of self-care for psychosocial clinicians. Participants identified their own mental well being as a precondition of being therapeutically effective. Maintaining emotional health was described as a continuous process requiring conscious effort, openness to learning, and personal growth. The capacity for self-reflection was identified as critical, allowing participants to learn from personal and professional experiences. In essence, the clinician brings aspects of “who they are” as individuals into the therapeutic encounter to achieve optimal therapeutic effectiveness. Some of the themes within this domain address work-specific opportunities (eg, *debrief with colleagues* and *value professional development*), whereas others speak to internal psychological issues (eg, *acknowledge your own feelings of vulnerability or helplessness*) or efforts to *maintain a balanced life*.

#### Domain B: Therapeutic approaches

This domain contains 16 themes that refer to various tasks, strategies, or techniques that clinicians use to help them communicate with and support patients experiencing distress. These themes describe a teachable skill set, comprised of various clinical practices and approaches. They are not hierarchical nor are they mutually exclusive, in that their application is wholly dependent on the specifics of the clinical encounter. For example, highly distressed and emotive patients may respond well to clinicians who help them *clarify and name the sources of distress* along with *problem solving*. Conversely, patients who are pensive or reticent to articulate their turmoil may respond to a therapeutic approach based on *probing for feelings underlying events and circumstances* or *acknowledging spiritual distress*.

#### Domain C: Creation of a safe space

This domain, comprised of 3 themes, underscores the importance of clinicians being able to *create a safe space* for clients (Fig. 1, domain C). The setting of therapeutic work must be a place in which patients feel safe and secure. It does not refer exclusively to a physical place but, rather, to a setting in which clinicians do their best to convey to patients that “this is their time.” Although an office or meeting room may be ideal, sometimes a drawn curtain or even physical proximity can be used to great effect to create a sense of intimacy and privacy.

### Hybrid Domains

During the iterative coding process, it became evident that many themes fit into more than 1 primary domain. For example, some themes appeared to describe aspects of *personal growth and self-care* (ie, domain A) but also fit under the rubric of *therapeutic approaches* (ie, domain B); as such, these items were placed within the overlap between them (Fig. 1, domain AB). Some themes overlapped at the interface between *therapeutic approaches* and the *creation of a safe space* (Fig. 1, domain BC), whereas others did so at the interface between *personal growth and self-care* and the *creation of a safe space* (Fig. 1, domain AC). These thematic hybrids were categorized according to this schema. On the basis of the constituent themes, overlapping domains were assigned the following labels: *therapeutic humility* (domain AB)*, therapeutic pacing* (domain BC), and *therapeutic presence* (domain AC).

#### Domain AB: Therapeutic humility

Certain themes are inseparable from *therapeutic approaches* and *personal growth and self-care*; that is, elements of both domains are intrinsic to the nature of these particular themes. Nine such themes were identified, collectively described and labeled as *therapeutic humility* (Fig. 1, domain AB). These themes address therapeutic strategies that implicate personal qualities and characteristics that clinicians need to be effective. For example, a strategy of *not avoiding emotion* and *being able to explore difficult topics* can be a very conscious therapeutic strategy, ie, not diverting the patient away from, or interfering with the engagement of, emotionally evocative and difficult material. However, this strategy demands that the clinician be able to tolerate and cope with significant emotional intensity. Similarly, *tolerating clinical ambiguity* speaks to the therapist's ability to cope with uncertainty. Allowing the clinical encounter to unfold in this way is a common therapeutic approach, providing patients the ability to explore a broad range of issues. However, it demands that therapists be comfortable taking a nondirective, nonauthoritarian stance. In general, themes subsumed within this hybrid domain underscore the need for therapists to be humble and nonpretentious, to acknowledge their patients' expertise, and to trust in the therapeutic process.

#### Domain BC: Therapeutic pacing

Seven themes overlapped between *therapeutic approaches* and *creating a safe space*. Collectively, these themes describe and are labeled *therapeutic pacing* (Fig. 1, domain BC). Each of these themes refers to a therapeutic strategy, which concurrently implicates the intensity or flow of the therapeutic process. The pace of a therapeutic session is an important element of creating a safe therapeutic space. Too slow a pace (ie, a pace that deals only with “here-now” issues and avoids exploring deeper content) can leave patients feeling frustrated or psychologically stuck. Conversely, a therapeutic pace that is too confrontational or emotionally evocative can cause patients to feel overwrought and overwhelmed. *Listening attentively*, *normalizing and validating the client experience and distress*, and *using skillful tentativeness* (ie, purposefully being hesitant and inquisitive to elicit disclosure and dialogue in a nonthreatening way) are all ways of promoting gently paced therapeutic engagement. Pacing must be tailored to suit the patient's specific needs and should be adjusted, moment by moment, within the therapeutic encounter.

#### Domain AC: Therapeutic presence

Eight themes were identified that resided at the interface between *personal growth and self-care* and *creation of a safe space*. These themes address personal qualities and attributes of the therapist, which directly contribute to and, thus, are indivisible from the sense of safety or security that patients experience as part of the therapeutic milieu. Collectively, these themes describe and were labeled *therapeutic presence* (Fig. 1, domain AC). Although it may seem intangible, *therapeutic presence* markedly shapes and informs the tone of care.^8^, ^35^ It requires clinicians to draw on deeply held personal qualities, such as *being compassionate and empathic*, *being respectful and nonjudgmental*, *being genuine and authentic*, *being trustworthy*, and manifesting these qualities in the service of making patients feel valued, affirmed, and understood.

Failure to evince these qualities can undermine patients' feelings of safety and comfort, threatening optimal therapeutic effectiveness. For example, failure to be *fully present* may cause patients to feel unworthy of their care provider's complete attention; failure to *value the intrinsic worth of the client* may cause patients to feel devalued or unimportant; and failure to *be emotionally resilient* may lead patients to withhold emotionally evocative material as a means of protecting the clinician.

#### Domain ABC: Optimal therapeutic effectiveness

In the initial analysis, no theme, in and of itself, was categorized within the domain ABC. Thus, according to the model, invoking any given theme, in the absence of others being brought into play, is unlikely to achieve optimal therapeutic efficacy.

### Model Validation

Participants attending the face-to-face workshops were provided an opportunity to critique the emerging model of therapeutic efficacy. For purposes of model validation, correct item categorization was understood to indicate concurrence with the initial primary or hybrid domain item placement (Table 3). Correct domain categorization meant that, although they did not agree with the exact item placement, they did agree with the overall domain placement (eg, instead of placing an item in domain A, they might have placed it in domain AC or AB; or, instead of placing an item in domain AB, they might have placed it in domain ABC). Item agreement exceeded 80% for all but 4 themes; *foster positive relationships between client and family*, *accept and honor client as expert*, *being mindful of boundaries*, and *offer comfort through touch*. Although the majority of participants believed that the theme *foster positive relationships between client and family* belonged in the primary domain *therapeutic approaches* (domain B), a minority believed it might be better located within 1 of the hybrid domains (domain AB, 7.8%; domain BC, 12.5%; domain ABC, 1.6%). Perhaps not surprisingly, *offer comfort through touch* was the most highly contested theme. Agreement was 67.2% on item categorization and 87.3% on domain categorization agreement. Although attempting to categorize components of therapeutic work is an exercise in deconstruction, a significant minority of participants believed that “touch” could not be dissociated from attributes typified within the domain of *therapeutic presence* (12.7% designated it as domain AC); others thought of it as consistent with a pacing technique, ie, providing calm, and consistent with a holding or grounding approach (11.1% designated it as domain BC). A minority of participants (4.7%), believed that *offer comfort through touch* was better classified with the domain of *therapeutic presence* (domain AC), ie, that it was qualitatively similar to other AC themes, such as compassion, empathy, respect, and trustworthiness.

**TABLE 3:** Validation of Model of Therapeutic Effectiveness

	Correct, %^a^	
	
Domains and Themes	Domain Categorization	Item Categorization
A: *Personal growth and self-care*		
1. Maintain a balanced life	100	100
2. Work at self-awareness	100	96.9
3. Acknowledge/work through our own fears	100	96.9
4. Acknowledge your own feelings of vulnerability or helplessness	100	98.4
5. Debrief with colleagues	100	93.8
6. Value professional development	NA	NA
B: *Therapeutic approaches*		
7. Clarify and name sources of distress	100	95.3
8. Problem-solve	100	100
9. Educate, inform client	98.4	93.8
10. Debunk myths	100	98.4
11. Reinforce client strengths and positive ways of coping	100	92.2
12. Provide techniques (eg, mindfulness, Therapeutic Touch)	100	98.4
13. Advocate for client with the care team	98.4	92.2
14. Foster positive relationships between client and family	100	78.1
15. Elicit client needs	98.4	96.9
16. Probe for feelings underlying events and circumstances	98.4	92.2
17. Help client identify what they can and cannot control	100	93.8
18. Help client understand by mirroring and reflection	98.4	85.9
19. Use silence to encourage client expression	98.4	82.8
20. Explore image and metaphor	100	95.3
21. Offer comfort through touch	87.3	67.2
22. Acknowledge spiritual distress	98.4	81.3
C: *Creation of a safe space*		
23. Provide privacy	100	98.4
24. Provide calming environment	100	89.1
25. Assure confidentiality	NA	NA
AB: *Therapeutic humility*		
26. Do not avoid emotion	100	100
27. Tolerate clinical ambiguity	100	93.8
28. Be able to explore difficult topics	96.9	96.9
29. Accept and honor client as expert	81.3	76.6
30. Be a catalyst for therapeutic change	93.8	90.6
31. Trust in the process	89.1	89.1
32. “Sit with” client emotional distress	92.2	87.5
33. Avoid urge to have to fix	92.2	92.2
34. Model healthy processing of emotion	93.8	93.8
BC: *Therapeutic pacing*		
35. Listen attentively	95.3	95.3
36. Hold or ground client	85.7	82.8
37. Keep client in the here and now	87.5	87.5
38. Maintain slow pace—do not rush therapy	89.1	87.5
39. Encourage client to talk about fear and distress	85.9	85.9
40. Normalize and validate client experience and distress	85.9	85.9
41. Use skillful tentativeness	90.6	90.6
AC: *Therapeutic presence*		
42. Being compassionate and empathetic	95.3	93.8
43. Being respectful and nonjudgmental	95.3	92.2
44. Being genuine and authentic	93.8	92.20
45. Being trustworthy	93.8	93.8
46. Being fully present	95.3	90.6
47. Valuing intrinsic worth of client	90.6	89.1
48. Being mindful of boundaries	76.6	76.6
49. Being emotionally resilient	85.9	85.9

Abbreviation: NA, not applicable.

a Note that correct item categorization indicates concurrence with the primary or hybrid domain item placement, and correct domain categorization indicates that, although there is disagreement with the actual item placement, there is agreement with the overall domain placement.

Participants were asked about the extent to which they endorsed the following 2 statements as part of the model-validation process: 1) using the model will enhance my ability to understand and address psychosocial distress with patients and clients, and 2) using the model will enhance my ability to teach others/students how to address psychosocial distress with patients and clients. For the former statement, 83% agreed or strongly agreed (42% and 41%, respectively); whereas. for the latter statement, 95% either agreed or strongly agreed (39% and 56%, respectively).

## DISCUSSION

The findings from this study are based on the collective insights and wisdom of a large number of knowledgeable and experienced psychosocial clinicians who care for patients with cancer. The nearly unanimous affirmation from workshop participants regarding the model's potential to inform practice and teaching strongly suggests that it has clinical and pedagogic applications. The model provides a lens through which any clinical encounters can be analyzed, critiqued, and better understood. For instance, although clinicians may attempt to *elicit patient needs* or *clarify and name sources of distress* (domain B themes), the performance of these tasks can fall short for many reasons: perhaps the patient feels that privacy has not been ensured or that the environment is simply too frenetic (domain C themes). Conversely, the patient simply may believe that the clinician is not paying complete attention and seems otherwise preoccupied or distracted (ie, lack of *being fully present* [domain AC]). Clearly, performing the seemingly appropriate clinical task does not equate, de facto, to an optimally executed therapeutic encounter. The model is dynamic and nonhierarchical, which means that not all constituent elements need to be applied in every situation; and health care providers will invoke those elements of therapeutic engagement that are most suitable based on individual patient characteristics and circumstances. However, health care providers and patients will be served better when the requisite primary domains of therapeutic effectiveness are considered in shaping their clinical encounters.

The current study has noteworthy limitations. The clinicians who volunteered for the study may represent a group of individuals who are particularly open to articulating their insights regarding the therapeutic process. However, they do represent a highly informed group of care providers with extensive professional experience. This study did not specifically focus on potentially negative therapeutic experiences.^36^ Rather, clinicians were tasked with identifying effective elements of therapeutic practice. Thus, the model is limited to explaining negative therapeutic outcomes on the basis of critical elements of practice across the various domains not being adequately invoked. The sample also was dominated by women and by social workers; ie, it could be argued that that our findings are biased toward a female and/or social-work perspective. However, differences regarding optimal therapeutic effectiveness based on sex or discipline were never raised during the course of detailed focus group discussions. Therefore, it appears that the identified core elements of therapeutic effectiveness are those that transcend considerations of sex and professional affiliation. Larger diverse samples may be required to examine differences in interprofessional responses.^37^

Despite vast progress in medicine, human nature remains unchanged. The need to be understood, the need for compassion and kindness—particularly in the context of deteriorating health—are, as they have always been, a core feature of our humanity. Various efforts have been made to understand the essential ingredients of how to comfort and assuage distress.^38^ Researchers are beginning to focus on issues such as therapeutic presence^35^, ^39^ and the integration of therapists' personal characteristics and learned techniques.^40^ Unlike other studies that have examined features of the therapeutic encounter,^25^, ^26^ the current study is unique by way of integrating various aspects of patient-health care provider interaction into an empirical model of therapeutic effectiveness. The insights gleaned from this study should help inform best practices and provide a structure and vernacular for therapeutic effectiveness, promoting the development of expertise and communication skills among clinicians caring for patients with cancer. It is conceivable that the model has implications and applications for health care providers beyond the realm of cancer. Therefore, future research is warranted, exploring how this model might influence the pedagogy of effective communication; determining its impact on patient distress; and, more broadly, accessing its ability to promote effective and humane medical care.
